# Programmable hyperspectral microscopy for high-contrast biomedical imaging in a snapshot

**DOI:** 10.1117/1.JBO.25.5.050501

**Published:** 2020-05-28

**Authors:** Jiao Lu, Yuetian Ren, Zhuoyu Zhang, Wenbin Xu, Xiaoyu Cui, Shuo Chen, Yudong Yao

**Affiliations:** a Northeastern University, College of Medicine and Biological Information Engineering, Shenyang, China; b Science and Technology on Optical Radiation Laboratory, Beijing, China; c Northeastern University, Ministry of Education, Key Laboratory of Data Analytics and Optimization for Smart Industry, Shenyang, China; d Stevens Institute of Technology, Department of Electrical and Computer Engineering, Hoboken, New Jersey, United States

**Keywords:** hyperspectral microscopy, multiplexed illumination, programmable optical filter, principal component analysis, linear discriminant analysis

## Abstract

**Significance:** Hyperspectral microscopy has been intensively explored in biomedical applications. However, due to its huge three-dimensional hyperspectral data cube, it typically suffers from slow data acquisition, mass data transmission and storage, and computationally expensive postprocessing.

**Aim:** To overcome the above limitations, a programmable hyperspectral microscopy technique was developed, which can perform hardware-based hyperspectral data postprocessing by the physical process of optical imaging in a snapshot.

**Approach:** A programmable hyperspectral microscopy system was developed to collect coded microscopic images from samples under multiplexed illumination. Principal component analysis followed by linear discriminant analysis scheme was coded into multiplexed illumination and realized by the physical process of optical imaging. The contrast enhancement was evaluated on two representative types of microscopic samples, i.e., tissue section and cell samples.

**Results:** Compared to the microscopic images collected under white light illumination, the contrasts of coded microscopic images were significantly improved by 41% and 59% for tissue section and cell samples, respectively.

**Conclusions:** The proposed method can perform hyperspectral data acquisition and postprocessing simultaneously by its physical process, while preserving the most important spectral information to maximize the difference between the target and background, thus opening a new avenue for high-contrast microscopic imaging in a snapshot.

## 1 Introduction

Hyperspectral microscopy technique integrates conventional microscopic imaging and spectroscopy methods to collect both spatial and spectral information of the sample.^1^ Spatially resolved biochemical information about the sample can be obtained,^2^ which promotes its widespread applications in biomedicine, ranging from fundamental research^3^^,^^4^ to clinical diagnosis.^5^^,^^6^ For those biomedical applications, the investigation of *in vivo* events is usually necessary, thus the hyperspectral microscopy system should exhibit an appropriate combination of high spatial, spectral, and temporal resolution.^7^ However, due to the inherent three-dimensional hyperspectral data cube with extremely large volume, hyperspectral microscopy technique typically suffers from slow data acquisition,^8^ mass data transmission and storage,^9^ and computationally expensive postprocessing.^10^

To overcome the above limitations, a programmable hyperspectral microscopy technique was proposed, which can capture coded microscopic images from a sample under coding illumination. The coding illumination was multiplexed with specific intensities at different wavelengths, leading to parameterized measurements of optical signals with different wavelengths. Such parameterized optical measurements can be equivalent to the results of hyperspectral data after numerical postprocessing, which enables hardware-based postprocessing by the physical process of optical imaging, i.e., coding numerical postprocessing in terms of the light source’s spectrum. Thus, hyperspectral data acquisition and postprocessing can be simultaneously implemented in a snapshot while preserving the most important spectral information to maximize the difference between the target and background. The proposed programmable hyperspectral microscopy technique was tested on two representative types of microscopic sample, i.e., tissue sections and cells. According to the results, the contrast of coded microscopic images was significantly improved compared to microscopic images collected under white light illumination.

## 2 Materials and Methods

In this study, two types of sample, i.e., longitudinal section of Cucurbita stem and osteoblast cells, were measured by the proposed programmable hyperspectral microscopy system. Conventional spectral scanning-based hyperspectral microscopic images were collected and subsequently postprocessed by principal component analysis and linear discriminant analysis (PCA–LDA) to differentiate target from background. Based on the PCA–LDA classifier obtained during the postprocessing, coding illumination can further be derived and then applied on the programmable hyperspectral microscopy system to capture a coded microscopic image (i.e., high-contrast microscopic image) in a snapshot. The coded hyperspectral microscopic images were compared with microscopic images collected under white light illumination, and the contrast enhancement was evaluated based on the average profile of the edge between target and background.

### 2.1 Programmable Hyperspectral Microscopy System

**Fig. 1:**
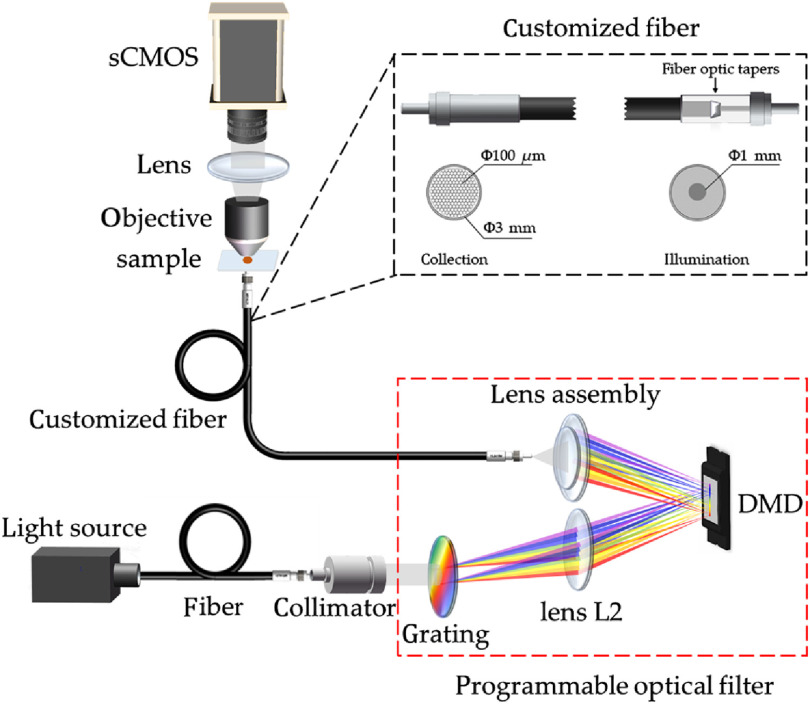
Schematic of the programmable hyperspectral microscopy system.

Figure 1 shows the schematic of the programmable hyperspectral microscopy system. White light from a xenon lamp (HSX-F300, NBET, China) was collimated and then passed through the programmable optical filter and a customized fiber to deliver multiplexed illumination of different wavelengths onto the sample. The programmable optical filter comprised a blazed transmission grating of 300 grooves/mm, an achromatic lens L2 with a focal length of 100 mm, a digital micromirror device (DMD) (DLP4500, Texas Instruments, United States), and a lens assembly (i.e., two achromatic lenses with focal lengths of 30 and 22 mm) in which the grating and DMD were placed at the focal planes on different sides of lens L2. More specifically, light with the same wavelength was dispersed in the same direction after passing through the transmission grating, and then focused onto the same column of micromirrors on the DMD after passing through lens L2. Calibration of the programmable optical filter was necessary and was performed using a compact spectrometer (FLA4000, Hangzhou Flight Technology Co., Ltd., China). More specifically, the programmable filter was modulated by setting one column of micromirrors on the DMD to the “on” state, while the other columns were set to the “off” state. The light from the xenon lamp first passed through the programmable optical filter and was then measured by the spectrometer in which the central wavelength, peak intensity, and full-width at half-maximum (FWHM) were recorded. The above procedure was repeated until all the columns of micromirrors on the DMD were calibrated once. Based on the above set up, the diffraction efficiency of the programmable optical filter was ∼39% in which the center wavelength can be finely tuned with the smallest step size of 0.3 nm and minimum FWHM of 12 nm from 400 to 676 nm. By modulating each micromirror on the DMD, i.e., setting each column of the micromirrors to on and off states, light with specific wavelengths can be refocused through the lens assembly into the customized fiber, thus multiplexed illumination of different wavelengths can be achieved. The multiplexed light, after interacting with the sample, was collected by a 50× objective lens (MPLN50X, Olympus, Japan) and an achromatic lens with a focal length of 30 mm, and then imaged onto the scientific CMOS (sCMOS) (Dhyana9, Tucsen, China).

The proposed programmable hyperspectral microscopic imaging system can be used to collect conventional hyperspectral microscopic images, coded microscopic images, and microscopic images under white light illumination. The conventional hyperspectral microscopic images were collected by spectral scanning method, also called staring or area scanning.^6^ More specifically, by setting several neighboring columns of the micromirrors on the DMD to the “on” state, monochromatic illuminations with specific wavelengths can be generated by white light passing through the programmable optical filter, thus narrow-band images under monochromatic illuminations with different wavelengths can be sequentially captured by sCMOS. The coded microscopic images were collected under coding illumination (i.e., specially designed multiplexed illumination) while performing continuous exposure on sCMOS. Furthermore, a microscopic image of the sample under white light illumination can be collected by setting all micromirrors to the “on” state, in which the light at all wavelengths can be reflected and delivered onto the sample.

### 2.2 Sample Preparation and Measurements

The longitudinal section of Cucurbita stem on a prepared slide was purchased from Bresser GmbH and directly used for imaging without further sample preparation. The osteoblast cells isolated from fetal rat calvaria were cultured in Dulbecco’s Modified Eagle Medium (01-052-1ACS, Biological Industries, Israel) supplemented with 10% fetal bovine serum (10270-106, Life Technologies, United States) and 1% penicillin–streptomycin (PS2004HY, Tian Jin Hao Yang Biological Manufacture Corporation, China) and were incubated in an incubator at 37°C and with 5% ${\mathrm{CO}}_{2}$. Cultures were maintained by the addition or replacement of fresh medium every 2 days until 80% of the bottom wall of the culture flask was covered with cells.

For both types of samples, conventional hyperspectral microscopic images, microscopic images under white light illumination, and coded hyperspectral microscopic images were taken by the proposed programmable hyperspectral microscopy system, respectively. For conventional hyperspectral microscopic images, 70 narrow-band images were collected from each sample by tuning the center wavelengths with a step size of 4 nm and an average FWHM of ∼12  mm from 400 to 676 nm. The exposure time for each narrow-band imaging was 3 s. Since the binary pattern rate of the DMD used in this study can reach up to 4225 Hz, the switching time of monochromatic illumination can be ignored and the total acquisition time for the hyperspectral microscopic images was 210 s. In order to remain consistent, the same exposure time (3 s) was applied on the acquisition of the coded microscopic image and the microscopic image under white light illumination. The coded hyperspectral microscopic image was taken from each sample under multiplexed illumination derived based on the PCA–LDA scheme as described in Sec. 2.3. The microscopic image under white light illumination was taken from each sample by setting all micromirrors to the “on” state. The contrast enhancement between the coded microscopic image and microscopic image under white light illumination was quantitatively evaluated based on the average profile of the edge between target and background from a single cell in a small region in which each profile consisted five pixels on each side of the edge and was normalized by the maximum intensity of the profile. More specifically, the contrast enhancement was quantitatively evaluated by the subtraction between the gradient coefficient of the coded microscopic image and that of the microscopic image under white light illumination in which the gradient coefficient referred to the decrease between the normalized intensities at both ends of the average edge profile.

### 2.3 Derivation of Coding Illumination

The region of interest (ROI) containing both target and background was selected from hyperspectral microscopic images in which the pixels within the ROI were manually cataloged into target and background groups. More specifically, the organelles inside the cell wall and the cell wall were treated as target and background for Cucurbita stem section, respectively, whereas the cells and the surroundings were treated as target and background for osteoblast cell samples, respectively. The sizes of the regions of interest were optimized based on the criterion that acceptable training classifiers can be built while minimizing the sizes of training samples. PCA followed by linear discriminant analysis was applied on those spectra to differentiate target from background in which the leave-one-out method^11^ was used for cross validation in an unbiased manner. The above PCA–LDA scheme was further used to derive coding illumination I, which can be calculated by the multiplication between the first k’th loading vectors $U_{1}$ and linear discriminant function $U_{2}$. More specifically, the first k’th loading vectors $U_{1}$ is equivalent to the first k’th constituent eigenvectors of the covariance D of hyperspectral data H after arranging eigenvectors from large to small according to the corresponding eigenvalues

$$D=H_{n}{H_{n}}^{T},$$ (1)

where $H_{n}$ is the normalized matrix of the hyperspectral data H and the superscript T represents the matrix transpose. Thus, $U_{1}$ is a n×k matrix in which n is the number of spectral bands (i.e., 70) and k is the number of principal component (PC) scores used in the above PCA–LDA scheme. The linear discriminant function $U_{2}$ is the eigenvector with the largest eigenvalue in the matrix of $M_{\sigma }M_{\tau }$. $M_{\sigma }$ and $M_{\tau }$ refer to the within-class scatter matrix and the between-class scatter matrix, respectively, and can be calculated according to the following equations:

$$M_{\sigma }=\sum \sum (s_{ij}-u_{i}){\left(s_{ij}-u_{i}\right)}^{T},$$ (2)

$$M_{\tau }=\sum (u_{i}-u){\left(u_{i}-u\right)}^{T},$$ (3)

where $s_{ij}$ refers to the j’th element of the i’th category’s principal eigenvalues, $u_{i}$ refers to the average of the i’th category’s principal eigenvalues, u is the average of all categories’ principal eigenvalues, and the principal eigenvalues are derived from the above PCA scheme. For the binary classification task, the linear discriminant function $U_{2}$ is a k×1 matrix. Thus, the coding illumination I is an n×1 matrix, and the optical measurements with such coding illumination were equivalent to the hyperspectral data after numerical postprocessing. To avoid negative values, the coding illumination I was further divided into imaging illumination $I_{i}$ and compensation illumination $I_{c}$, in which the coding illumination is actually the subtraction between imaging illumination and compensation illumination. Therefore, the coded microscopic image R can be obtained by the subtraction between the microscopic images under imaging illumination $R_{i}$ and compensation illumination $R_{c}$, as shown in the following equation:

$$R=HU_{1}U_{2}=HI=HI_{i}-HI_{c}=R_{i}-R_{c}.$$ (4)

More detailed deduction of coding illumination derivation can also be found in our previous study.^12^

## 3 Results and Discussions

**Fig. 2:**
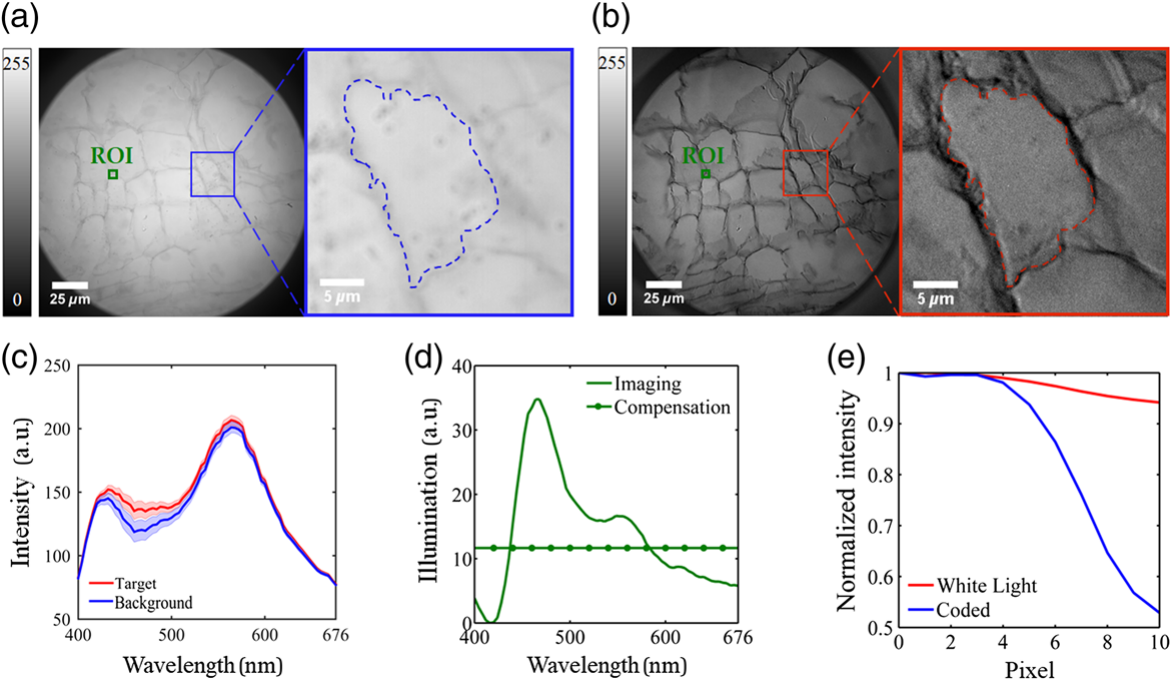
The results from Cucurbita stem section: (a) microscopic image under white light illumination, (b) coded microscopic image under coding illumination, (c) average spectra of target and background within ROI, (d) the spectra of imaging illumination and compensation illumination, and (e) the normalized average profile of the edge between target and background in the coded microscopic image and microscopic image under white light illumination.

For the Cucurbita stem section, an ROI with 53×54  pixels was selected for further PCA–LDA classification and coding illumination derivation, as shown in Fig. 2(a). More specifically, the spectrum at each pixel within the ROI was treated as a single sample for further postprocessing. The first four PCs were employed and a classification accuracy of 90.53% was achieved with PCA–LDA strategy by cross validating the above ROI data set. Based on the PCA–LDA classifier obtained above, the coding illumination can be derived and further divided into the imaging illumination and compensation illumination to avoid negative intensities, as shown in Fig. 2(d). According to Fig. 2(d), the spectral information about differentiating the target from background was dominated by wavelengths ranging from 416 to 420 nm and 448 to 492 nm; better contrast hyperspectral microscopic images as well as larger spectral differences between target and background were consistently observed within such wavelength ranges. The resulting coded microscopic image is shown in Fig. 2(b), and significant contrast enhancement can be observed compared to microscopic image under white light illumination as shown in Fig. 2(a). The image contrast enhancement was further quantified by the average profile of the edge between target and background from a single cell, in which the organelles’ inside cell wall and the cell wall were manually segmented. According to Fig. 2(e), the normalized intensities along the average edge profile drop much more sharply for the coded microscopic image, especially from the fourth to the tenth pixels, and the image contrast was enhanced by ∼41%. By further applying a threshold of 90, the proposed method can reach a classification accuracy of 81.8% within the ROI.

**Fig. 3:**
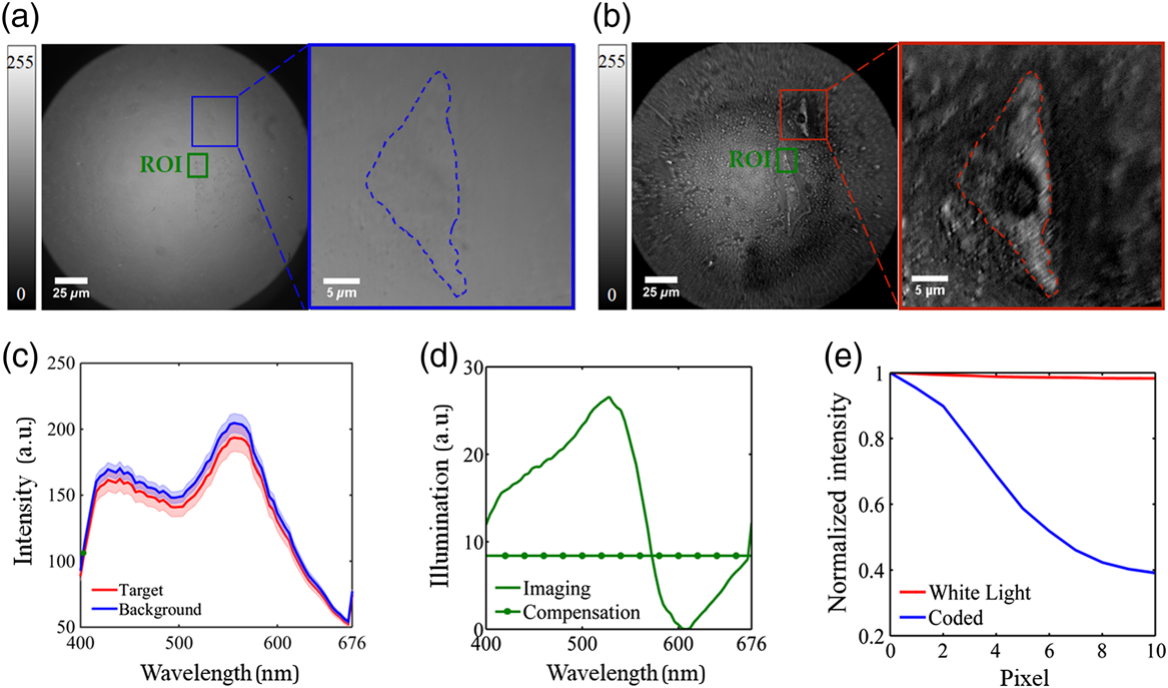
The results from osteoblast cells: (a) microscopic image under white light illumination, (b) coded microscopic image under coding illumination, (c) average spectra of target and background within ROI, (d) the spectra of imaging illumination and compensation illumination, and (e) the normalized average profile of edge between target and background in the coded microscopic image and microscopic image under white light illumination.

For osteoblast cells, an ROI with 178×121 pixels was selected for further PCA–LDA classification and coding illumination derivation, as shown in Fig. 3(a). The larger ROI for the osteoblast cell sample can be attributed to the fact that the variance of the training spectra is larger and the difference between the target and background spectra is smaller for the osteoblast cell sample compared to those for the Cucurbita stem section. The spectrum at each pixel within the ROI was treated as a single sample for further postprocessing. The first four PCs were employed and a classification accuracy of 80.4% was achieved with PCA–LDA strategy by cross validating the above ROI data set. Based on the PCA–LDA classifier obtained above, the coding illumination can be derived and further divided into the imaging illumination and compensation illumination to avoid negative intensities, as shown in Fig. 3(d). According to Fig. 3(d), the spectral information about differentiating the target from background was dominated by wavelengths ranged from 440 to 556 nm, in which better contrast hyperspectral microscopic images as well as larger spectral differences between target and background were consistently observed within such a wavelength range. The resulting coded microscopic image is shown in Fig. 3(b), and significant contrast enhancement can be observed compared to the microscopic image under white light illumination as shown in Fig. 3(a). The image contrast enhancement was further quantified by the average profile of the edge between target and background from a single cell, in which the osteoblast cell and surroundings were manually segmented. According to Fig. 3(e), the normalized intensities along the average edge profile drop much more sharply for the coded microscopic image, and the image contrast was enhanced by ∼59%. By further applying a threshold of 115, the proposed method can reach a classification accuracy of 70.4% within the ROI.

The significant improvement of the contrast can be attributed to the employment of the PCA–LDA scheme during the physical process of optical imaging, in which PCA can compress the most spectral information about the sample into a few PC scores^13^ and LDA can maximize the between-class distance.^14^ Thus the coded microscopic image is actually the weighted accumulation of optical signals at different wavelengths based on the PCA–LDA scheme using coding illumination. More specifically, the optical signals of wavelengths with larger target-background difference were intensified, whereas the optical signals of wavelengths with smaller target-background difference were alleviated, thus high-contrast microscopic image can be captured in a snapshot. Since the PCA–LDA scheme was employed, it is expected that the background can be completely removed by applying a proper threshold in an ideal case.

Compared with conventional hyperspectral microscopic imaging techniques, the proposed method can perform hyperspectral data acquisition and postprocessing simultaneously in a snapshot, in which the time-consuming hyperspectral data acquisition and subsequent postprocessing can be replaced by the collection of a single coded image. Since only a single coded image is necessary, the proposed method can speed up the acquisition by dozens of times, alleviate storage space and transmission time, and avoid computationally expensive postprocessing, while maintaining the most critical spectral information for target identification, thus opening a new avenue for high-contrast microscopic imaging in a snapshot. Although the coded image in this study was obtained by subtraction between the microscopic images under imaging illumination and compensation illumination, it is due to the negative parameters induced by the PCA–LDA scheme. This problem might be solved by using some advanced methods, such as non-negative PCA^15^ and non-negative matrix factorization.^16^ Compared with other DMD-based hyperspectral imaging techniques,^17^ the major difference is that DMD in our method is functioned as a wavelength scanner to deliver multiplexed illumination onto the sample, which enables hardware-based hyperspectral data postprocessing by the physical process of optical imaging in a snapshot. Furthermore, by only coding the PCA scheme into programmable hyperspectral microscopy system (i.e., coding the first several PC loading vectors into multiplexed illuminations), the three-dimensional hyperspectral data cube can be compressed and then recovered from several coded images by spectral reconstruction algorithms,^18^^,^^19^ and the signal-to-noise ratio can be significantly improved because of the integration of intensities along the wavelength dimension,^20^ thus offering an alternative way for hyperspectral microscopic imaging with high spatial, spectral, and temporal resolution. The major drawback of the proposed method is that the system needs to be recalibrated and training data set needs to be rebuilt if any of the optical components are changed, such as the light source. However, a simple solution is to measure the spectral responses of the original system and the new system with a spectrometer and then compensate the spectral response difference by modulating the transmittance of the proposed programmable optical filter.

## 4 Conclusions

In this study, a programmable hyperspectral microscopy technique based on multiplexed illumination was proposed and investigated, in which a PCA–LDA scheme can be coded into multiplexed illumination and realized by the physical process of optical imaging. Thus, hyperspectral data acquisition and postprocessing can be simultaneously implemented in a snapshot, while preserving the most important spectral information to enhance the contrast. According to the results of tissue section and cells, the contrast of coded microscopic images was improved significantly compared to the microscopic image collected under white light illumination; thus the proposed method offers a new solution for high-contrast microscopic imaging in a snapshot.
